# Depletion of the SR-Related Protein TbRRM1 Leads to Cell Cycle Arrest and Apoptosis-Like Death in *Trypanosoma brucei*

**DOI:** 10.1371/journal.pone.0136070

**Published:** 2015-08-18

**Authors:** Gabriela V. Levy, Carolina P. Bañuelos, Analía G. Níttolo, Gastón E. Ortiz, Nicolás Mendiondo, Georgina Moretti, Valeria S. Tekiel, Daniel O. Sánchez

**Affiliations:** 1 Instituto de Investigaciones Biotecnológicas, Universidad Nacional de San Martín (IIB-UNSAM)—Consejo Nacional de Investigaciones Científicas y Técnicas (CONICET), 25 de Mayo y Francia. Gral. San Martín, Buenos Aires, Argentina; Instituto Butantan, Laboratório Especial de Toxinologia Aplicada, BRAZIL

## Abstract

Arginine-Serine (RS) domain-containing proteins are RNA binding proteins with multiple functions in RNA metabolism. In mammalian cells this group of proteins is also implicated in regulation and coordination of cell cycle and apoptosis. In trypanosomes, an early branching group within the eukaryotic lineage, this group of proteins is represented by 3 members, two of them are SR proteins and have been recently shown to be involved in rRNA processing as well as in pre-mRNA splicing and stability. Here we report our findings on the 3rd member, the SR-related protein TbRRM1. In the present study, we showed that TbRRM1 ablation by RNA-interference in *T*. *brucei* procyclic cells leads to cell-cycle block, abnormal cell elongation compatible with the nozzle phenotype and cell death by an apoptosis-like mechanism. Our results expand the role of the trypanosomal RS-domain containing proteins in key cellular processes such as cell cycle and apoptosis-like death, roles also carried out by the mammalian SR proteins, and thus suggesting a conserved function in this phylogenetically conserved protein family.

## Introduction

The protozoan parasite *Trypanosoma brucei* is the causative agent of sleeping sickness in humans, which remains a public health problem in sub-Saharan Africa, and the related disease “Nagana” in cattle. Sleeping sickness threatens millions of people in 36 countries, who live in remote areas with limited access to adequate health services, hampering the surveillance and therefore the diagnosis and treatment of cases [1].

*T*. *brucei* belongs to the order Kinetoplastida and has a complex life cycle alternating between the tsetse fly and a mammalian host [2]. Its life cycle is characterized by a series of differentiation steps resulting in stages that differ morphologically, structurally and biochemically. Moreover, these stages alternate between non-replicative and replicative forms, indicating a well coordinated control between differentiation and cell cycle [3]. As in typical eukaryotic cells, *T*. *brucei* follows the G0/G1, S, G2 and M phases along its cell cycle. However, this parasite possesses particular features like the absence of mitosis to cytokinesis checkpoint in the procyclic stage [4,5] that leads to the appearance of anucleated cells termed zoids [6] when parasites are arrested in the G1/S or M phases [4,7–9]. *T*. *brucei* contains a number of single-copy organelles and cytoskeletal structures, which need to be accurately duplicated and segregated prior to cell division. Therefore, precise regulation of organelle segregation is essential to ensure a correct cell division (for review see [10]), which underscore a tight association between control of morphogenesis and cell cycle progression [5]. For instance, G1/S arrested cells have been associated to abnormally elongated posterior end described as “nozzle phenotype” [11]. Previous reports have shown that modifying the levels of different proteins can induce this phenotype. Among them, RNAi of proteins involved in cell cycle regulation such as the cyclin Cyc-2 [12] and ALBA proteins [13] or the double knockdown of the cdc2-related kinases CRK1-CRK2 [14] as well as overexpression of two members of the CCCH zinc finger family [11,15] have been shown to produce nozzle cells.

As in higher eukaryotes, deregulation of the cell cycle in trypanosomatids is one of the pathways that leads to apoptosis-like cell death. In mammalian cells, SR proteins have been implicated in linking such processes. For instance, in cell-cycle arrested mammalian cells, the ASF/SF2 splicing factor promotes expression of pro-apoptotic splicing variants through an alternative splicing network that leads to apoptosis, thus showing how cell cycle-arrested cells orchestrate the apoptotic response [16]. In trypanosomes, proteins involved in the cell cycle, such as the cyclin-dependent kinase CRK3 and centrin, which regulates cytokinesis, have been shown to be linked to apoptosis-like cell death (for review see [17]).

Gene expression in trypanosomatids includes some unusual features such as RNA editing, polycistronic transcription of protein-coding genes and transcription of certain pre-mRNAs by RNA Pol-I [18]. In addition, virtually all the pre-mRNAs are processed to mRNAs via *trans*-splicing coupled to polyadenylation [19–21]. Genes belonging to the same polycistronic unit generally do not encode for functionally related proteins and, indeed, mRNAs from adjacent genes show differences either in abundance or in the stage-specific expression in the parasite. It is therefore believed that gene expression in kinetoplastids is regulated mainly post-transcriptionally through its 3’ UTR at the level of mRNA degradation and translation [22,23] or by regulating *trans*-splicing [21,24]. In recent years, proteomic analysis has brought considerable progress to the identification and characterization of trypanosome spliceosomal proteins such as Sm [25], U1 [26], SMN and Gemin2 proteins [27]. An important group of splicing factors is the SR protein family, whose characteristic members possess one or two N-terminal RNA recognition motifs (RRM) and a C-terminal domain rich in serine/arginine dipeptides, the RS domain [28]. SR proteins have been extensively studied in eukaryotes given their relevance in key cellular processes like constitutive and alternative splicing of pre-mRNA and processes beyond splicing such as nonsense-mediated mRNA decay and various stages of metabolism of the mRNAs, such as export, stability and translational regulation [28,29].

*T*. *brucei* posses a total of 75 RRM proteins [30]; three of them belong to the SR or SR-related protein family (TSR1, TSR1IP and TRRM1). TSR1, was previously implicated in *trans*-splicing [31], whereas its ortholog in *T*. *cruzi*, TcSR, was shown to participate in *cis*-splicing in a heterologous system [32]. More recently, Gupta et al. [33], showed that TSR1 and TSR1IP [34] are involved in mRNA stability and rRNA processing in addition to splicing regulation.

TRRM1 (TriTrypDB acc no: Tb927.2.4710, also known as TbRRM1), a *T*. *brucei* SR-related protein, was proposed that it might be, somehow, implicated in the coordination of the events involved in its complex replication cycle although no data was presented to suggest that TbRRM1 is directly implicated in the parasite cell cycle [35]. Recently, TbRRM1 was shown to be associated to numerous mRNAs, which suggests a role in post-transcriptional regulation. In addition, it has also been postulated that it may play a role in maintaining a permissive chromatin to facilitate transcription and RNA processing [36]. A genome-wide RNAi target sequencing screen showed that TbRRM1 is essential in both procyclic and bloodstream forms [37]. Previously, we demonstrated that the *T*. *cruzi* ortholog of TbRRM1, named TcSR62 (acc no: TcCLB.511621.50) is relocated to the nucleolus when parasites are exposed to transcriptional stress or severe heat shock, a behavior shared with other RBPs involved in mRNA metabolism [38] and the bulk of poly(A)+ RNA [39]. Interestingly, neither RBPs nor poly (A)+ are relocated to the nucleolus in *T*. *brucei* [40].

In the present study we demonstrate that *TbRRM1* silencing in procyclic cells (PC) leads to cell growth inhibition and cell death by arresting cells at the G1/S phase of the cell cycle, which is then followed by an apoptotic-like process. We also show the emergence of cells exhibiting aberrant nucleus-kinetoplast configuration suggesting compromised post-mitotic nuclear positioning events. Finally, we identified TbNOP86 as a target of TbRRM1, whose mRNA level is strongly down-regulated after *TbRRM1* silencing.

## Methods

### RNA-interference plasmid construction

Tetracycline inducible p2T7Ti vector [41] was used to knockdown *TbRRM1* (Tb927.2.4710) expression in *T*. *brucei* PC, strain 427 pLew13 pLew29, also named 29–13 [42]. The sequences corresponding to 5'UTR and ORF region of the TbRRM1 transcript were amplified from genomic DNA by PCR using the following primers: 5’ UTR region (forward primer 1: 5'-CGTCTAGAGCGTTAGTCTGTCGGACGTT-3’ and reverse primer 2 5'-CGTCTAGAGTTACACCGTACCCACCACC-3'); ORF region: (forward primer 3: 5’-GAGTCTAGAGAAAAGACGACGGAGGATGT-3’ and reverse primer 4: 5’-CGTTCTAGAAACCAACAAAGGCGAACATC-3’). The PCR amplified fragments were cloned into pGEM-Teasy from Promega, digested with XbaI, and subcloned into an XbaI-digested p2T7Ti.

### Cell cultures and transfections

*T*. *brucei* PC, strain 29–13 were transfected and maintained in SDM-79 (Life Technologies) cell culture media as described previously [43]. Cell density was determined daily using a Neubauer haemocytometer. When necessary, hygromycin at a final concentration of 50 μg/ml, G418 at 10 μg/ml and phleomycin at 2.5 μg/ml were added (all from Invivogen). Independent clones were generated by limiting dilution cloning. Unless otherwise indicated, to induce RNAi in procyclic forms, the cells were grown to a density of 5x10^6^ cells/ml, then diluted to a density of 1x10^6^ cells/ml, and maintained in absence or presence of 1 μg/ml tetracycline (TET, from Invivogen). Cell densities were determined every 24 h and cumulative growth curves were plotted taking into account the dilution factors necessary to maintain cultures below a density of 1×10^7^ cells/ml.

### Northern blot analyses

For Northern Blot analyses, parasites were grown until 72 h continuously. Twenty micrograms of total RNA were electrophoresed on formaldehyde agarose gels (1.5%) and blotted by capillary onto Zeta Probe nylon membranes (Bio-Rad). Cross-linked membranes were blocked in hybridization buffer (3X SSC, 1% BSA, 7% SDS and 1 mM EDTA) for 4 h at 60°C and hybridized overnight with radioactive probes at 60°C to 65°C. DNA probes were prepared by PCR in presence of α^32^P-dCTP. Membranes were washed three times with decreasing concentrations of SSC (3X, 1.5X and 0.5X) for 15min at 62°C. The TbRRM1 probe was amplified with forward primer (5'-GGATCCATGCAACAATATACCCTTCG-3') and reverse primer (5'-GGATCCTCGCTGACGCCTCTCAAT-3'). The 28S rRNA (Tb427.02.1975) probe was amplified with forward primer (5’-GTAGTATAGGTGGAAGCGCAAG-3’) and reverse primer (5’-CCAGCTCACGTTCCCTGTCA-3’). Genes ID and sequences were obtained from TriTrypDB, http://tritrypdb.org/tritrypdb/).

### Western blot analyses

Total protein was recovered from 6.5x10^6^ un-induced or induced parasites grown continuously until 72 h. Whole cell extracts were electrophoresed on an SDS-PAGE (12% gel) and transferred to ECL membrane (GE Healthcare); Anti-TbRRM1 mouse antiserum was used at 1:1000 dilution. Either anti-transaldolase (TAL) or anti-enolase (Enol) rabbit sera were used as loading control. Peroxidase-labeled goat anti-mouse or goat anti-rabbit (both from Thermo Scientific) were used as secondary antibodies. SuperSignal West Pico from Thermo Scientific was used as chemiluminescent substrate.

### Cell cycle distribution by FACS analyses

For DNA content determination a total 5x10^6^ cells from asynchronous un-induced or induced cultures were harvested at 300xg for 10min, and washed twice in cold phosphate-buffered saline (PBS)-2 mM EDTA. Cell pellets were resuspended in 300 μl PBS-2 mM EDTA and mixed with 700 μl of cold absolute methanol and kept overnight at 4°C. Cells were then washed twice with PBS-2 mM EDTA at 4°C and resuspended in 500 μl of staining solution (950 μl PBS-2mM EDTA, 20 μl RNase A 10 mg/ml, and 50 μl propidium iodide (PI) 1 mg/ml and incubated for 30min at 37°C. The DNA content of PI stained cells was analyzed in FL-2 by Partec CyFlow Space cytometer. The percentages of cells in each phase of the cell cycle (G1, S, and G2/M) were calculated using FlowJo software (Tree Star Inc.).

### Phosphatidylserine exposure assay

A total of 5x10^6^ cells from un-induced or induced cultures were harvested at 300xg for 1min, washed once with PBS and resuspended in 1 ml of binding buffer (10 mM HEPES, 140 mM NaCl, 2.5 mM CaCl_2_, 10 mM glucose, pH: 7.4). Cells were then aliquoted in 200 μl and stained with FITC-annexin V and PI (BioLegend) according to the manufacturer's protocol. The samples were analyzed by FACS and visualized under a Nikon Eclipse E600 microscope.

### BrdU incorporation assay

Cells were incubated with 200 μM 5-bromo-2-deoxyuridine (BrdU) and an equimolar amount of 2-deoxycytidine (both from Sigma) at 28°C for 2 h and then harvested by centrifugation at 300xg, washed once in cold PBS, fixed with 70% cold methanol ON at 4°C, and handled for FACS analysis or indirect immunofluorescence (IIF) as described earlier with minor modifications [44]. Briefly, for IIF assay 4% PFA fixed-parasites were settled onto poly-L-lysine-coated slides and DNA was denatured by adding 4 N HCl for 45min at room temperature. Slides were washed three times in 0.1 M borate buffer pH: 8.5 for 5min and once in PBS to remove the acid and then incubated for 90min at room temperature in a moist atmosphere with monoclonal anti-BrdU antibody (Sigma) diluted 1:500 with PBS, pH: 7.4, 0.5% Tween 20 (Sigma) and 0.5% bovine serum albumin (BSA, Sigma). After washing, samples were incubated with 1:1000 Alexa-Fluor 488-conjugated anti-mouse (Molecular Probes) diluted in PBS containing Tween 20 and 1.5% normal goat serum (Sigma) and incubated for 1 h at room temperature. Then cover slides were washed and mounted in antifade reagent (FluorSave, Calbiochem) containing 1 μg/ml 4,6-diamidino-2-phenylindole (DAPI, from Life Technologies), observed under a microscope (Nikon Eclipse 80i) using appropriate fluorescence emission filters, and photographed. Labeling of parasites for FACS was performed similarly to IIF-handled parasites. Data were acquired with a Partec CyFlow Space cytometer, and analyzed with FlowJo software. Alterations in the fluorescence intensity of BrdU incorporation were quantified from BrdU positive population, by the index of variation (IV) obtained by the equation (TM-CM)/CM, where TM is the mean of FL-1 fluorescence for TET+ parasites and CM is that of the non-treated cells.

### Mitochondrial membrane potential and cell viability assays

Cell viability assays and detection of mitochondrial membrane potential were performed using Rhodamine 123 (Rho123) staining from Life Technologies. Parasites from un-induced or TET induced cultures were harvested at 300xg for 10min, washed in cold PBS and then resuspended in PBS-2 mM glucose. Cells were incubated with 10 μg/ml of Rho123 in PBS for 15min at 28°C and then washed with cold PBS-2 ml glucose. Fluorescence intensities of Rho123 in cells were analyzed by flow cytometric analysis using FL-1 detector. For detecting non viable cells, 1 μg/ml propidium iodide was added 5 minutes before FACS analysis. A total of 100.000 events were acquired in the region previously established as corresponding to *T*. *brucei* PC, based on the forward (FSC) and side (SSC) scatter. For mitochondrial membrane potential assay, percentage of cells with decreased Rho123 relative intensity was obtained excluding the region of FL-1 positive cells from 24h TET- samples.

### Immunofluorescence assays

Cells were harvested, and deposited on poly-L-lysine-coated coverslips. After washing twice with PBS, cells were fixed in 4% paraformaldehyde (PFA) in PBS. Cells were permeabilized with 0.5% saponin. To detect the TbRRM1 protein, parasites were incubated with rabbit antiserum diluted 1:1000 for 1 h at room temperature. Alexa Fluor 488-conjugated goat anti-rabbit (Molecular Probes) was used as secondary antibody. Tyrosinated tubulin was detected with YL1/2 mouse monoclonal antibody (Serotec, UK) diluted 1:1000, and an Alexa Fluor 594-conjugated goat anti-rat (Molecular Probes) was used as secondary antibody. Finally, the coverslips were mounted using FluorSave reagent containing 1 μg/ml DAPI. Cell samples were examined under a Nikon Eclipse E600 microscope.

### Morphometric analysis and Nucleus-Kinetoplast (N-K) configuration studies

For morphometric analysis, parasites were fixed with 4% PFA and stained with 1 μg/ml DAPI before (0 h) or after 24, 48 and 72 h TET addition. Nucleus-Kinetoplast configuration, parasite morphologies and distances from Nucleus (N) to Kinetoplast (K), as well as from K to posterior end (P) were evaluated. Parasites were examined under Nikon 80i LED microscope and images were obtained from more than 200 parasites for each time point treatment and analyzed by ImageJ software. Parasites were considered as nozzled cells were K-P distance was greater than 4.4 μm (evaluated as mean K-P distance plus 2xSD in 24 h TET- cultures).

### RNA isolation and RTqPCR

Total RNA was isolated from un-induced and induced parasites with Trizol Reagent according to manufacturer's instructions (Life Technologies) and RNA integrity was evaluated by 1% agarose gel electrophoresis. Samples were incubated with RQ1 DNAse (Promega) followed by chloroform extraction and ethanol precipitation. First strand cDNA was synthesized from total RNA samples using Superscript II reverse transcriptase (Life Technologies). Briefly, 3 μg RNA were combined with 200 ng of random primers, 0.5 mM of each dATP, dCTP, dTTP and dGTP, denatured at 65°C for 5 min and quickly chilled on ice. Final 20 μl reactions were assembled with final concentrations of 1X First Strand Buffer, 10 mM DTT and 200 U Superscript II reverse transcriptase. Reactions were incubated at 42°C for 50 min and then 70°C for 15 min. Real time quantitative PCR (qPCR) was performed using Kapa Sybr Fast Universal Kit (Biosystems) with primers described below. TbRRM1 mRNA abundance was measured with the oligonucleotides F 5’-CAATGGGACGTATGGGACAT-3’ and R 5’-TCGTCTGTCACCACCCATAG-3’. TbNOP86 mRNA was measured with oligonucleotides F 5’-TTTTTGACCGTGGTGGGAGG-3’ and R 5’-TTGATCCCTTCTCACAGCGG-3’, TbActin-A (Tb927.9.8850) with primers F 5’-GTACCACTGGCATTGTTCTCG-3’ and R 5’-CTTCATGAGATATTCCGTCAGGTC-3’. The relative quantity of 18S rRNA (Tb427.02.1931) using the oligonucleotides-F 5’-ACGGAATGGCACCACAAGAC-3’ and R 5’-GTCCGTTGACGGAATCAACC-3’, was used as endogenous control. The reactions were carried out with the 7500 Real Time PCR System from Applied Biosystems. The results were analyzed using the standard curve method and normalized to 18S rRNA.

### Statistical analyses

All data were expressed as means ± SD of at least three independent experiments and graphics were performed using the GraphPad Prism 4.0 software. Data were analyzed by Student t test compared to 24 h TET- results.

## Results

### TbRRM1 silencing by RNAi arrests cell growth and affects cell morphology

**Fig 1:**
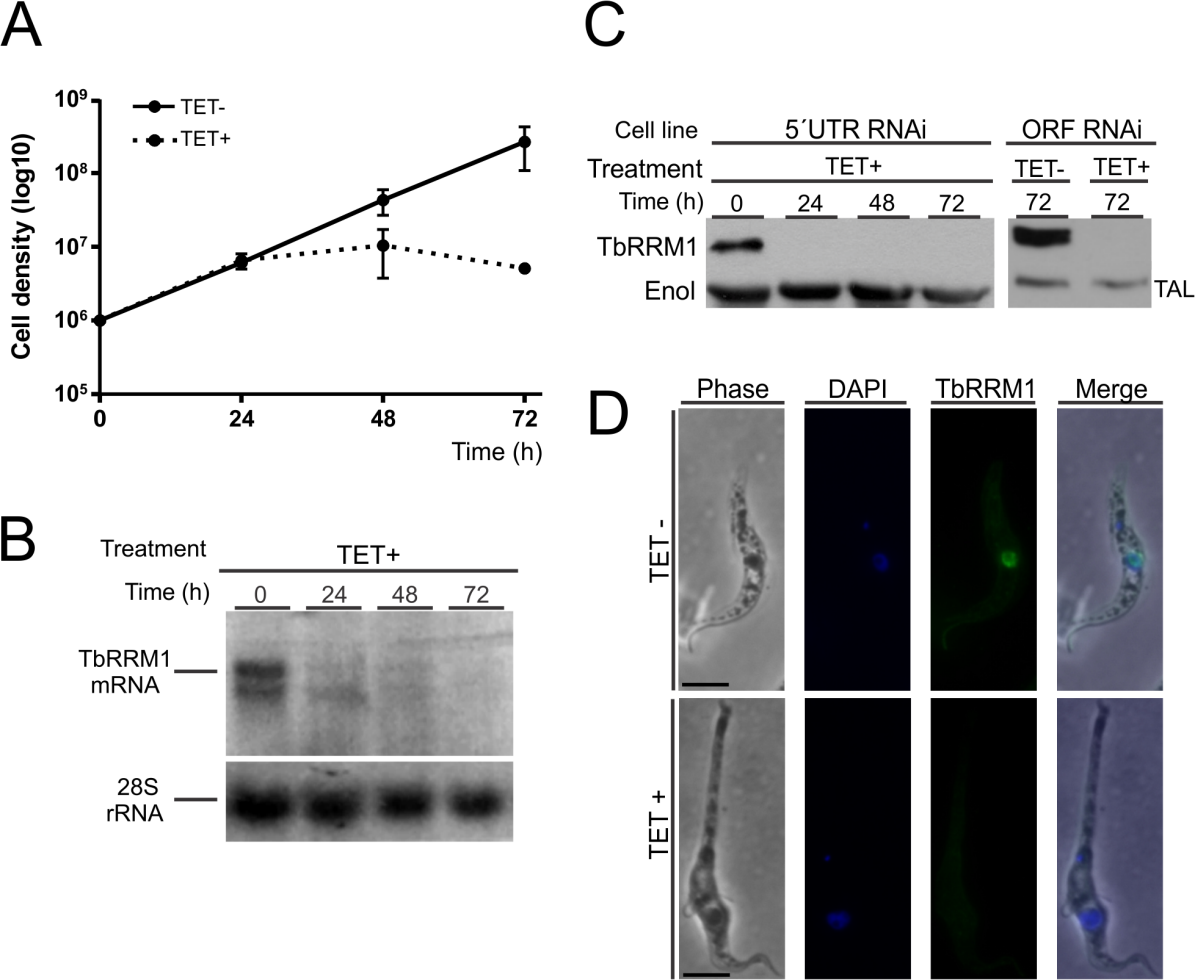
TbRRM1 is essential for parasite survival. **(A)** Cumulative growth of TbRRM1 RNAi trypanosomes in the absence (TET-) or presence (TET+) of tetracycline for the indicated times. Error bars indicate the standard deviations around the means of three independent experiments. **(B)** Northern blot analysis of TbRRM1 mRNA after silencing. A 28S rRNA probe was used as loading control. **(C)** Western blot analysis of TbRRM1 over time following induction of TbRRM1 RNAi in both 5´UTR and ORF cell lines. Either anti-transaldolase (TAL) or anti-enolase (Enol) serum was used as loading control. **(D)** Immunofluorescence of TbRRM1 in un-induced cultures or 48 h after TET addition. From left to right: phase contrast, DAPI stained cells, anti-TbRRM1 stained cells and merged images. Note the nozzle phenotype in TET+ cells.

It has been reported that *TbRRM1* knockout cells displayed a perceivable phenotype in which the majority of cells become generally enlarged, misshapen, anucleated or vacuolated [35]. In order to elucidate the role of TbRRM1 in cell shape and proliferation, a RNAi approach was taken. For this purpose a fragment of 443 bp corresponding to the 5’UTR region of the *TbRRM1* gene was cloned into the tetracycline inducible p2T7^Ti^ vector [41] and transfected into 29–13 *T*. *brucei* PC. After selection, transfectants were cloned by limiting dilution and one clone was selected for further analysis. Growth curves were determined in the absence (TET-) or presence (TET+) of tetracycline to induce TbRRM1 double-stranded RNA. Induction of TbRRM1 RNAi resulted in almost complete growth arrest after 24 h, while un-induced cells continued unaffected their exponential growth phase (TET+ and TET-, respectively, Fig 1A). Despite the growth arrest, the parasites remained viable during the first 48 h. However, after 72 h post induction, cell viability was strongly compromised as shown by viability assays (S1 Fig). To demonstrate that this outcome was associated with a specific down regulation effect, TbRRM1 mRNA levels were determined by northern blot analysis. Fig 1B shows that TbRRM1 mRNA levels decreased significantly after 24 h of RNAi induction. To further corroborate this result, we performed a western blot analysis to determine the TbRRM1 protein level after TET addition. After 24 h induction, TbRRM1 was undetectable (Fig 1C, left panel) indicating a nearly complete RNAi penetrance. In addition, microscopic analysis of stained cells with anti-TbRRM1 antibody (Fig 1D, TbRRM1 panels) showed a clear nuclear labeling in un-induced cells (TET- panels), whereas in induced cells (TET+ panels) the label was completely absent. Interestingly, the microscopic analysis also showed that a large proportion of induced cells presented an abnormal morphology with a markedly long posterior end (Fig 1D, phase contrast panels).

To rule out any off-target effect, a second RNAi construct was made targeting a region within the *TbRRM1* ORF (bases: 217 to 739). Similar inhibitory effects on growth and protein expression levels (Fig 1C, right panel) and phenotypic changes of cells carrying any of the two RNAi constructs were observed.

### TbRRM1 deficient cells exhibit a nozzle phenotype

**Fig 2:**
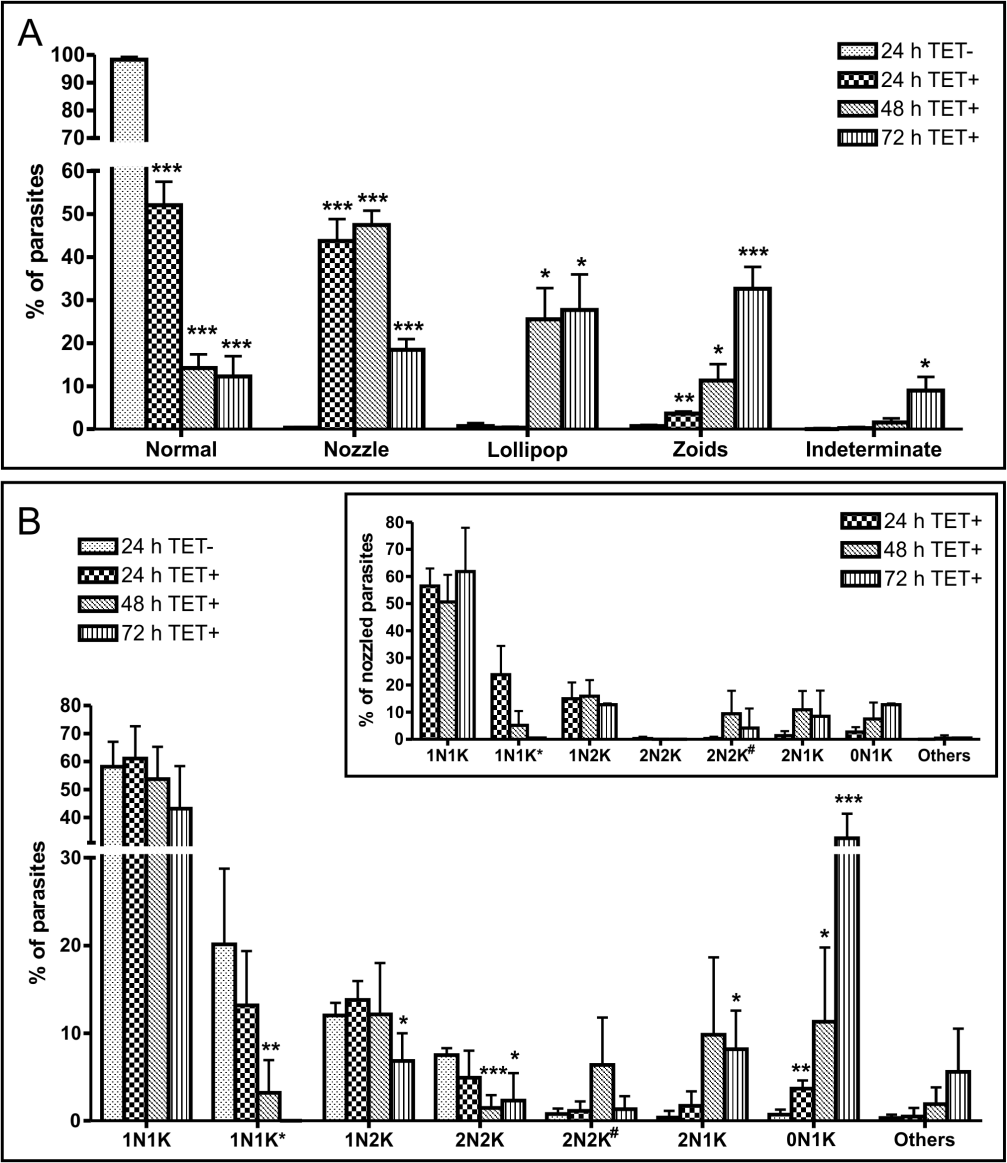
TbRRM1 silencing induced aberrant nucleus-kinetoplast (N-K) configurations and abnormal phenotypes. **(A)** Distribution of normal and aberrant phenotypes (nozzle, lollipop, zoid and indeterminate cells) after RNAi induction. **(B)** N-K configuration graphics showing the percentage of cells presenting xNxK arrangement at different time points after RNAi induction. Inset: Percentage of N-K configuration of the nozzled cells from 24 h to 72 h after TET addition. For reference, 1N1K* indicates cells with one nucleus—one elongated kinetoplast; 2N2K# represents parasites with abnormal N-K distribution. Results were obtained from more than 200 cells at each time point of three independent experiments. Data was analyzed by Student t test compared to 24 h TET- values. *p<0.05; **p<0.01; ***p<0.001.

**Fig 3:**
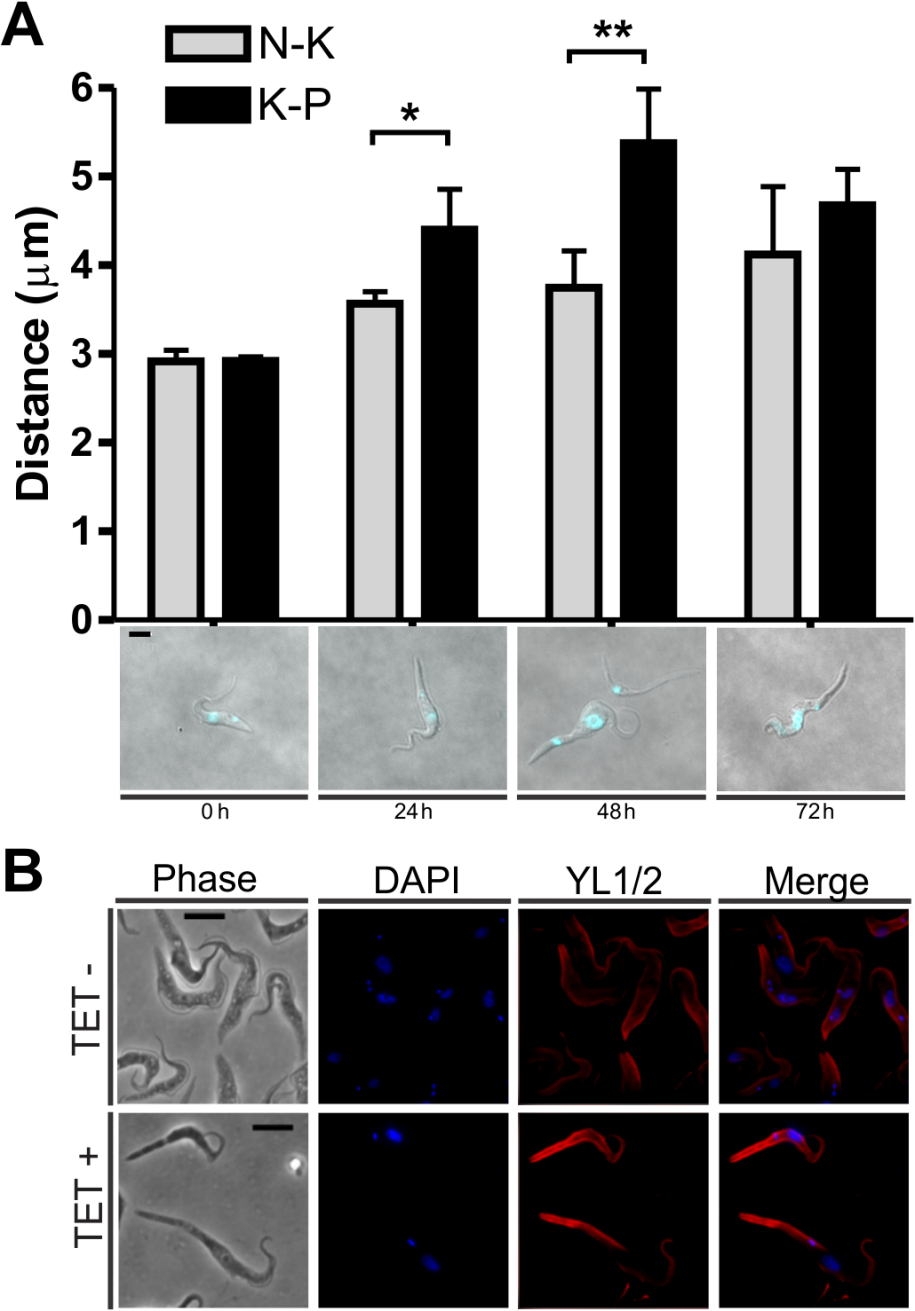
TbRRM1 deficient cells exhibited a nozzle phenotype. **(A)** Upper: Graphic bars showing the length from nucleus to kinetoplast (N-K; gray bars) or from kinetoplast to posterior end (K-P; black bars) at 0, 24, 48 and 72 h post RNAi induction. Results are expressed as mean ± standard deviation from more than 200 cells at each time point of three independent experiments (Student t test. *p<0,05; **p<0,01). Bottom: DAPI stained PC. **(B)** Cytoskeleton analysis of 48 h *TbRRM1* silenced cells by immunofluorescence (anti-tyrosinated tubulin YL1/2). From left to right: phase contrast, DAPI stained cells, YL1/2 stained cells and merged images. Scale bar: 3 μm.

The morphological changes associated to the induction of *TbRRM1* silencing (Fig 1D) were examined in more detail. To this end, TbRRM1 RNAi cells were induced or not with TET and cell morphology was scored as shown in Fig 2A. Parasites with normal morphology decreased significantly after a 24 h of treatment, dropping to about 12–15% after 48–72 h, whereas cells with an enlarged posterior end (nozzled cells) increased to 40–50% of the total population after 24–48 h of treatment and then dropped to about 20% by 72 h (Fig 2A). The anucleated cells (zoids) increased continuously and presented different morphological subpopulations (S2 Fig, panels A-C), whose proportion changes along the growth curve (S2 Fig, panel E). The subpopulations of rounded cells presenting a cytoplasmic extension, which were dubbed “lollipops” (S2 Fig, panel D), and indeterminate cells increased significantly after 48 h of TbRRM1 depletion. The elongated cells were reminiscent of the nozzle phenotype originally described by Hendriks et al. [11], which is characterized by an enlarged posterior end of G1-arrested PC. To investigate whether enlarged cells were indeed expressing the nozzle phenotype, we carried out morphometric studies by measuring the distance from nucleus to kinetoplast (N-K) and from kinetoplast to posterior end (K-P) in un-induced (0 h) and induced (24–72 h) parasites. In un-induced cells both distances increased over time, but remained approximately equal (Fig 3A). However, in induced parasites the K-P distance increased significantly over the N-K distance at 24 and 48 h of TET addition (Fig 3A). We then analyzed the outgrowth of the microtubule cytoskeleton, which is also characteristic of the nozzle phenotype [11]. The staining of cells with YL1/2 antibody, which recognizes tyrosinated alpha-tubulin present in newly assembled microtubules [45], showed that TET+ cells presented stronger YL1/2 intensity at the extended posterior end than TET- cells (Fig 3B). Taken together, both analyses support the notion that elongated cells induced by depletion of TbRRM1 in PC display the nozzle phenotype.

### TbRRM1 silencing induces aberrant Nucleus-Kinetoplast configurations

Control of morphogenesis and cell cycle progression are tightly connected in *T*. *brucei* [5]. As it has been reported that cells displaying the nozzle phenotype are mainly arrested in the G1/S phase of the cell cycle [11,14], we investigated whether TbRRM1 ablation affects the cell cycle progression tabulating cells with different N-K configuration [46]. As previously described, cells presenting 1N1K arrangement are in G1 and S phases, while those with 1N2K are in the G2 phase and cells with 2N2K configuration are mitotic or post-mitotic [47]. Within the 1N1K population, cells containing an elongated kinetoplast were scored separately as 1N1K*, such cells belong mostly to the nuclear S phase [47,48]. We also scored cells with aberrant N-K configuration such as zoids (0N1K, anucleated cells, containing a single kinetoplast) and 2N1K cells (Fig 2B). The simultaneous presence of both configurations within a parasite population is an indication that some parasites were compromised in either mitosis or post-mitotic nuclear positioning, but still able to complete cell division [4]. In addition, 2N2K cells were categorized as cells having a normal (2N2K) or abnormal (2N2K^#^) N-K distribution.

Induction of TbRRM1 RNAi resulted in a significant decrease of cells exhibiting 1N1K* configurations after 48 h of TET addition (Fig 2B), which suggests a G1/S arrest. The normal 2N2K population also decreased significantly after 48 h of treatment, which correlates with an increase in the number of abnormal 2N2K^#^ cells, thus suggesting an incorrect repositioning of the cell nuclei after mitosis. 2N2K^#^ cells are probably the progenitors of 2N1K and 0N1K cells [6,49], both of which rise significantly from 48 h post induction. However, taken into account both the significant increase of zoids 24 h before the rise of 2N1K cells, and the larger number of zoids relative to 2N1K cells at 72 h, points to an additional progenitor of zoid parasites, most probably the 1N2K cells. As shown before, following inhibition of the nuclear S-phase, 1N2K PC do not enter mitosis, but can continue with cell division to generate 1N1K and 0N1K cells [4].

The subset of cells exhibiting an obvious nozzle phenotype was also analyzed separately to score N-K configuration. Surprisingly, the nozzle population presented both normal and abnormal N-K configurations as shown for the total population (Fig 2B, inset). This result suggests that nozzled cells generated by TbRRM1 depletion are not G1/S cell-cycle arrested. However, as also seen in the population as a whole, the number of nozzled 1N1K* cells decreased significantly after 48 h of TbRRM1 depletion, which suggests an arrest at the G1/S phase, but intriguingly without a concomitant accumulation of 1N1K parasites. Interestingly, all 2N2K nozzled cells displayed an abnormal N-K arrangement (2N2K^#^), which correlated with the number of 2N1K and 0N1K nozzled cells generated.

### TbRRM1 silencing induces cell cycle arrest

**Fig 4:**
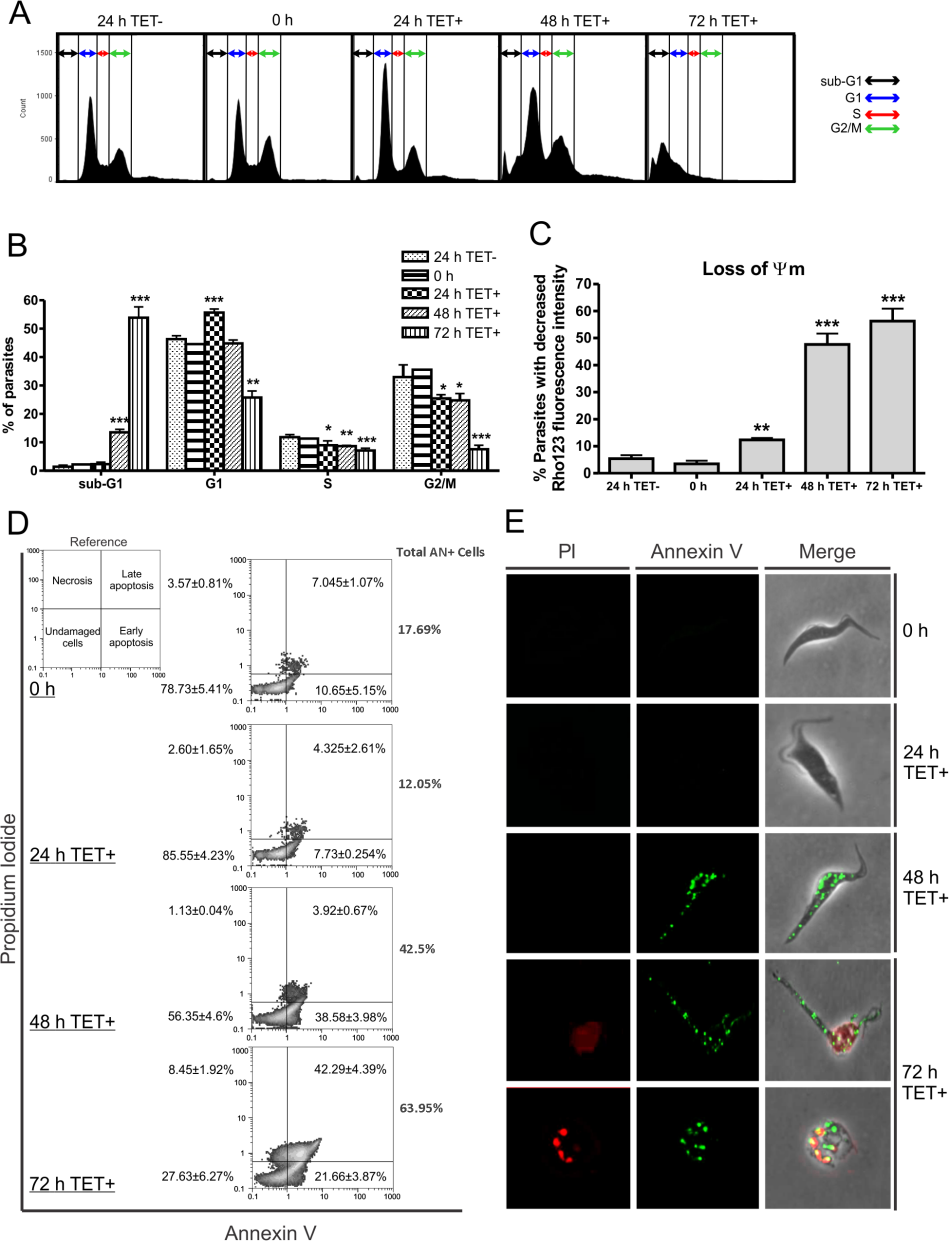
TbRRM1 silencing induced cell cycle arrest and apoptosis. **(A)** FACS profiles of propidium iodide (PI)-stained cells after RNAi induction (TET+) are shown as FL-2 histograms. Both, cells sampled at 24 h without RNAi induction (24 h TET-) which corresponds to growth from an equivalent starting density to the 24 h RNAi induced cells and cells taken prior induction (0 h) are displayed as un-induced controls. **(B)** Graphic bars indicating the percentage of cells in each cell cycle phase after RNAi induction. **(C)** Loss of mitochondrial membrane potential evaluation by Rho123 staining. Graphic bars showing the percentage of apoptotic-like parasites with decreased fluorescence intensity at different time points after TET addition. **(D)** Analysis of Annexin V and PI stained cell populations by FACS after *TbRRM1* silencing. **(E)** Fluorescence microscopy of Annexin V and PI stained cells after RNAi induction. Data were analyzed by Student t test compared to 24 h TET- values. *p<0.05; **p<0.01; ***p<0.001.

As shown before, depletion of TbRRM1 induced morphological changes that are compatible with the nozzle phenotype (Fig 3) and aberrant N-K configurations that suggest alterations in mitotic or post-mitotic nuclear events (Fig 2B). However, analysis of the cell cycle by N-K arrangements did not show accumulation of G1 cells (a sign of blocked G1/S transition), something we were expecting because of the known association of the nozzle phenotype with arrest in G1/S [11,14]. To further analyze the cell cycle, we determined the DNA content of induced and un-induced TbRRM1-RNAi cells by flow cytometry (FACS) (Fig 4A and 4B, see also S1 Table). After 24 h of TET addition, the percentage of cells in G1 increased ~9% relative to 24 h un-induced cells (from 46.34% to 55.65%). This increase was coupled with a decrease in the percentage of cells in S- as well as in G2-phase (from 11.82% to 8.98% and from 32.98% to 25.45%, respectively). FACS results also indicated that at 48 h post-induction the proportion of cells in the G1 phase of the cell cycle decreased relative to 24 h after TET addition. This fact was probably due to cell death since the sub-G1 population increased significantly from 2.3% (24 h TET+) to 13.5% (48 h TET+), reaching almost 54% of the population on day 3 (72 h TET+, Fig 4B). This broad peak of sub-G1 cells is characteristic of cells that undergo necrosis or programmed cell death [50], which include DNA fragmentation and loss of DNA fragments.

Together, these results suggest that TbRRM1 ablation disturbed normal cell cycle progression by arresting cells at the G1 phase after 24 h of TET addition (Fig 4A and 4B), a time point that is coincidental with the inhibition of the cell proliferation (Fig 1A). After further 24 h of TbRRM1 depletion, the cells seemed to initiate an apoptotic-like process leading to cell death.

### TbRRM1 depletion leads to DNA synthesis impairment

**Fig 5:**
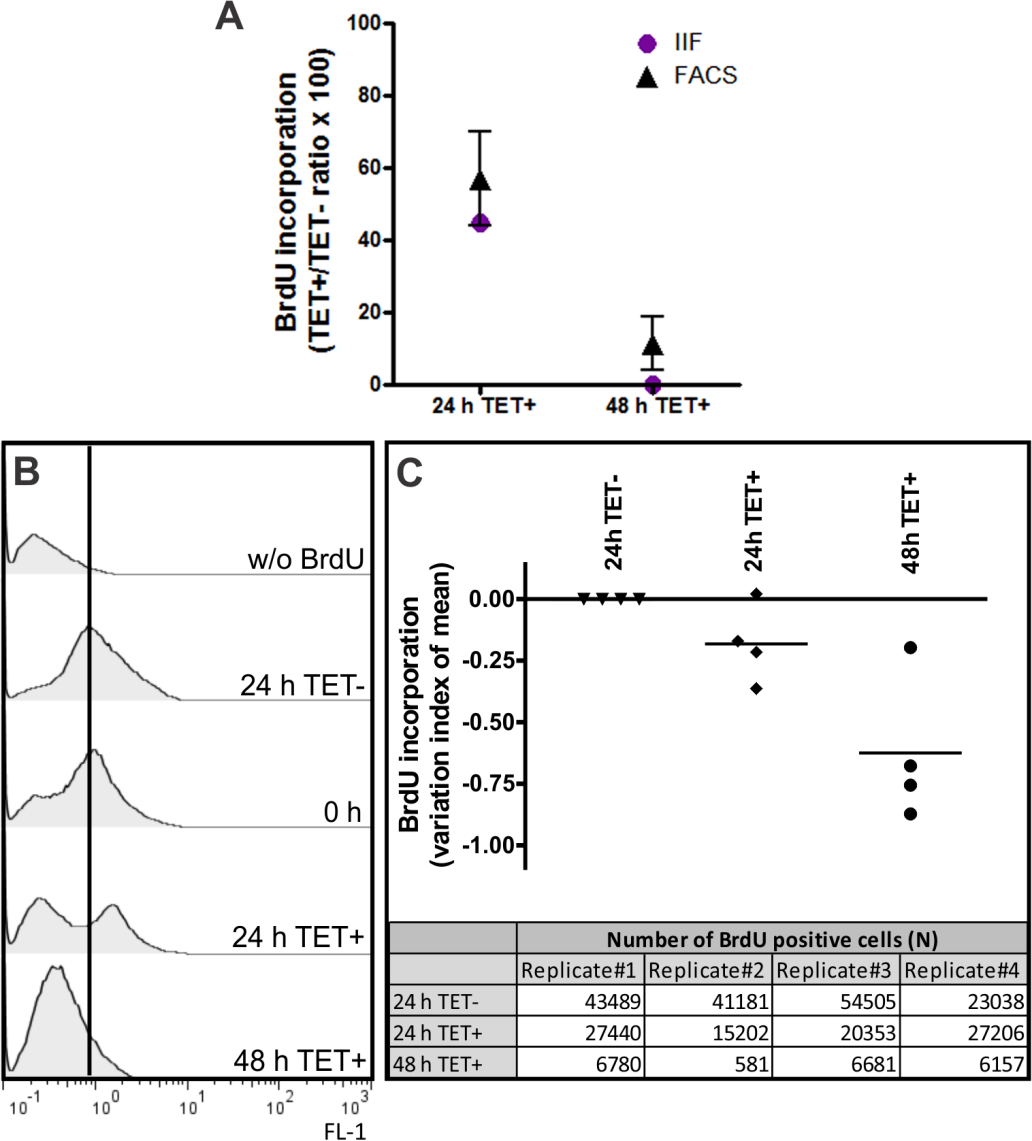
TbRRM1 depletion induced impairment of DNA synthesis. **(A)** BrdU incorporation assay over time obtained by immunofluorescence (IIF, circles) or FACS (triangles). DNA synthesis impairment was determined by the TET+/TET- ratio, calculated as the percentage of BrdU-positive cells from both 24 h and 48 h TET+ cultures relative to the percentage of BrdU-positive cells from 24 h and 48 h TET- cultures, respectively, multiplied by 100. Error bars indicate the standard deviations around the means of the TET+/TET- ratio from either four independent experiments for FACS assay or three for IIF. **(B)** FACS histograms of un-induced or TET+ treated parasites labeled with BrdU (FL-1). Histograms representative of quadruplicate experiments are shown. **(C)** Graphic showing the index of variation (IV) obtained by the equation (TM-CM)/CM, where TM is the mean of FL-1 fluorescence for TET+ parasites and CM is that of the non-treated cells. Horizontal line shows the IV mean for the biological replicates. The table indicates the number of BrdU positive cells analyzed in each independent experiment.

FACS analysis (Fig 4A and 4B) showed a modest increase (~9%) of G1 cells after 24 h of TbRRM1 RNAi induction. If parasites are truly arrested in the G1 phase then there should be a concomitant decrease in nuclear DNA synthesis. However FACS analysis showed a small, although significant decrease of cells in the S phase. To further analyze the DNA synthesis, we determined the percentage of cells that incorporated bromodeoxyuridine (BrdU) into their DNA by indirect immunofluorescence (IIF) and by FACS (Fig 5). Both analyses demonstrated that BrdU incorporation was compromised in induced cells. FACS results (Fig 5A and S3 Fig) indicated that the number of cells synthesizing DNA dropped ~43% and ~89% after 24 h and 48 h of RNAi induction, respectively. Similar results were obtained by IIF analysis (Fig 5A and S4 Fig, panel A).

These results suggest that TbRRM1-depleted cells were indeed cell cycle arrested at the G1/S phase since the number of cells that incorporated BrdU, that is traversing the S-phase, dropped significantly from 24 h post induction compared to control cells. In addition, it also suggests that DNA synthesis was compromised in *TbRRM1* silenced parasites since FACS analysis also showed a time-dependent decrease of the BrdU fluorescence intensity (Fig 5B and 5C).

### TbRRM1-deficient cells undergo apoptosis-like death

To further characterize the apoptotic/necrotic process observed after 72 h of TbRRM1 depletion, un-induced (24 h TET- and 0 h) and induced (24–72 h TET+) TbRRM1 RNAi cells were stained with Annexin V (AN) and propidium iodide (PI) and analyzed by FACS. After 48 h of induction, AN+ cells, but PI- increased from 10.65% to 38.58%, while the AN+PI+ population remained approximately constant (4–7%) (Fig 4D). However, after 72 h, the AN+PI+ population increased to 42.29% and the PI+ cells increased to ~50% of the population (Fig 4D). These data indicate that ~40% of the cells depleted of TbRRM1 for 48 h were stained only with AN, a sign of early apoptosis and that most of these cells also became permeable to PI 24 h later, suggesting that TbRRM1-depleted cells initiate an apoptotic-like cell death process after 48 h of silencing. PI and AN stained cells were also visualized by fluorescence microscopy. Nozzled cells displayed staining characteristic of early apoptosis, AN+ PI-, at 48 h after RNAi induction (Fig 4E). At 72 h post-induction, most of the cells became deformed and showed clear signs of late apoptosis such as AN+ PI+ staining (Fig 4E, 72 h panels). Additionally, some cells showed condensation of the chromatin or fragmentation of the nucleus, which are also characteristics of apoptotic processes [17] (Fig 4E, bottom panels).

To further refine these findings, we analyzed the mitochondrial membrane potential as it is well known that mitochondrial membrane potential loss is a hallmark for apoptosis. RNAi induced and un-induced PC were stained with Rhodamine 123 (Rho123), which is an aromatic cationic dye that distribute itself into the mitochondrial matrix in response to the mitochondrial membrane potential. As shown in Fig 4C the percentage of cells with a decrease in the membrane potential increased significantly after TET addition reaching 47.6% and 56.23% of the cells at 48 h and 72 h post induction, respectively. Moreover, alterations in the mean relative fluorescence also indicated that *TbRRM1* silencing led to mitochondrial membrane potential loss in a time-dependent manner (S5 Fig). These results are compatible with the previous experiment and, together, suggest that the deficiency of TbRRM1 in PC promotes cell death by an apoptotic-like process.

### The expression of the nucleolar protein TbNOP86 is regulated by TbRRM1

**Fig 6:**
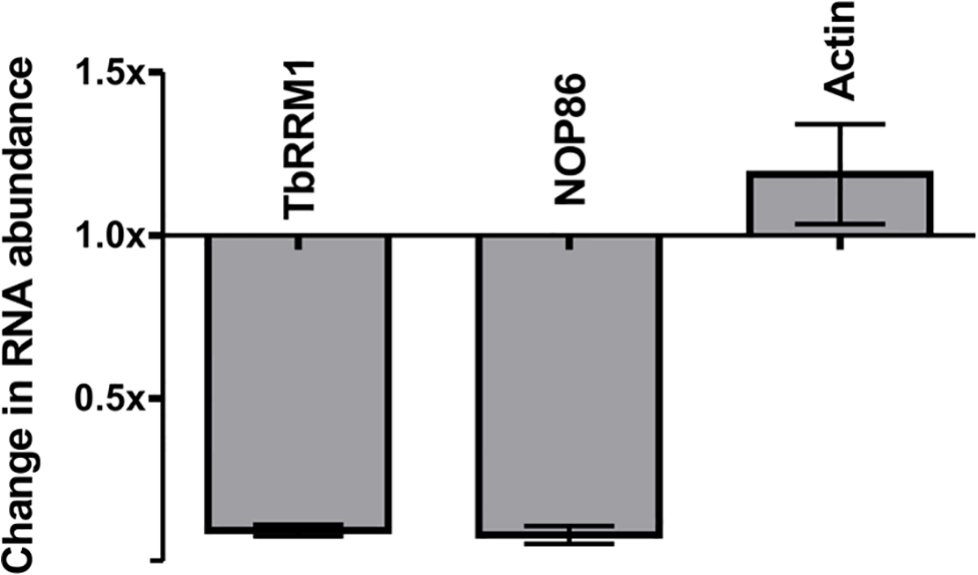
TbRRM1 is involved in the regulation of TbNOP86. Change in mRNA abundance after 24 h of TbRRM1 RNAi induction assayed by RTqPCR. Values are expressed as mRNA relative abundance from TET+ / TET- assays. Data were obtained from at least three independent experiments.

Recent studies performed by Naguleswaran et al (2015) [36] demonstrated the down regulation of numerous transcripts after TbRRM1 depletion, in agreement with our previously unpublished results. The gene coding for the nucleolar protein TbNOP86 (acc no: Tb09.160.1160), was one of the genes whose mRNA level decreased significantly after depletion of TbRRM1. To confirm this finding, the mRNA abundance of TbNOP86 was tested by RTqPCR in parasites depleted or not of TbRRM1. Actin-A and TbRRM1 were included in these assays as controls. The results shown in Fig 6 demonstrate that the mRNA abundance of TbNOP86, was significantly reduced after TbRRM1 RNAi induction, decreasing to a similar level as TbRRM1, while no change was detected in the mRNA level of actin-A.

## Discussion

In the present work, we show that TbRRM1 depletion induces growth arrest, morphological changes, aberrant N-K configurations, cell cycle block and cellular death by an apoptotic-like process in *T*. *brucei* PC.

Morphological analyses of TbRRM1 depleted cells showed that the absence of TbRRM1 in procyclic parasites elicits important morphological changes, which is in agreement with the previously reported by Manger and Boothroyd [35]. Induced cultures showed a prevalence of nozzled cells (~45–48%) during the first two days of treatment, which are then gradually replaced mainly by zoids and by rounded cells, being the last ones probably dying apoptotic-like cells.

Analysis of N-K configurations showed that two aberrant cell populations arose after 48 h of TbRRM1 RNAi induction: one presenting a 0N1K configuration (zoids) and the other a 2N1K configuration. Both cell types, probably sibling cells generated from cell-cycle compromised 2N2K cells, have been related to altered mitosis or post-mitotic nuclear positioning [6,49]. Interestingly, among the nozzled cells we also observed abnormal, 2N1K and 0N1K, as well as normal N-K configurations (Fig 2B, inset), suggesting that a proportion of the nozzled cells bypassed the cell-cycle arrest, or the commencement of nozzle formation precedes the G1/S block, and hence the inhibition of DNA replication [12]. In agreement with these results, 24 h after RNAi induction some nozzled cells were also able to synthesize DNA (S4 Fig, panel B). However, after further 24 h of TbRRM1 depletion, ~80% of the cells, both normal and nozzled, were unable to replicate their DNA. Regarding to the nozzle formation and cell-cycle phase, previous results from other labs have shown that overexpression of *TbZFP2* in PC results in the nozzle phenotype, all of them restricted to the early phase of the cell cycle (1N1K) [11]. On the contrary, nozzled cells generated by expressing a RNAi against the trypanosome cyclin CYC2, displayed all type of normal N-K configurations (1N1K, 1N2K and 2N2K) [12]. Our findings show, in addition to normal configuration, the presence of 2N1K and 0N1K cells among the nozzled population. This result suggests that these cells are doubly affected: first, by uncontrolled microtubule extension at the posterior end of the cells leading to the nozzle phenotype, and second, by altered post-mitotic nuclear positioning giving rise to both 2N1K and zoid cells. Interestingly, within the nozzle population all 2N2K cells displayed abnormal nucleus distribution (Fig 2B, inset) suggesting that in these cells the nozzle formation and abnormal nucleus arrangement are linked and might be a consequence of the same defect.

Analysis of BrdU incorporation (Fig 5) clearly showed an impairment of DNA synthesis starting 24 h after TbRRM1 depletion. This fact is also supported by the decrease of 1N1K* cells (Fig 2B), which represent cells in the nuclear S phase of the cell-cycle. Both results support the notion of a cell cycle arrest at the G1/S phase induced by depletion of TbRRM1. In addition, it seems that also the DNA synthesis is impaired in cells that enter into the S phase (Fig 5C). This finding correlates with the increased number of zoids, relative to 2N1K cells, which might originate from nuclear S-phase inhibited 1N2K cells [4].

In addition to cell cycle arrest, both abnormal morphology and N-K configuration, TbRRM1 depletion also induced an apoptotic-like cell death process. This last conclusion is supported by i) the exposure of phosphatidylserine in the outer leaflet of the plasma membrane, a sign of early apoptosis, demonstrated by the Annexin V assay (Fig 4D). At 48 h post-induction, ~40% of the cells were stained with Annexin V, but not with PI (characteristic of necrotic cells); ii) a significant increase of cells with a sub-G1 content of DNA (Fig 4A and 4B), suggestive of cells with degraded DNA, iii) maintenance of an intact plasma membrane until late stages of the apoptotic process showed by PI staining of non-permeabilized cells (S1 Fig); and, iv) the decrease of the mitochondrial membrane potential of the cells at 48 and 72 h post induction (Fig 4C). Apoptosis-like death has already been described in trypanosomatids and can be induced by different stresses and deregulation of essential pathways and biological processes such as the cell cycle [17,51]. Like in higher eukaryotes, in trypanosomes cell cycle control and apoptosis seem to be linked (reviewed in [17]). Unsurprisingly, it has been shown that kinases are important players linking both processes [5]. The results here presented, showing that TbRRM1 is involved in cell cycle control and apoptosis-like death, strongly suggest that also RNA binding proteins might be key players in the regulation of both processes.

Deregulation of the trypanosome microtubule cytoskeleton has also been shown to be associated with apoptosis-like death [17]. In mammalian cells, such a link is mainly associated with cell-cycle arrest in M phase and signaling pathways such as the mitotic spindle assembly checkpoint activation [52]. In *T*. *brucei*, the nucleolar protein TbNOP86 is required for mitotic progression, as its depletion leads to accumulation of G2/M cells [53]. In procyclic forms, this is accompanied by an increase in both zoids and 2N1K cells due to the absence of the mitosis to cytokinesis checkpoint in this parasite stage [6]. As shown here (Fig 6), depletion of TbRRM1 induced a significant decrease of the TbNOP86 mRNA level from 24 h post induction; so it is tempting to speculate that the emergence of cells with abnormal N-K configuration is a consequence of TbNOP86 depletion. Taken into account these data, it seems plausible that TbRRM1 depletion is also related-although indirectly- to a cell cycle arrest in late mitosis.

Altogether, TbRRM1 probably regulates the trypanosome cell cycle in at least two main points, one at the G1/S phase through as yet unknown effector(s) and the other at the post-mitotic phase through the expression regulation of TbNOP86. Arrest of the cell cycle at either or both phases may acts as a strong trigger of the apoptotic pathway.

Recently, Naguleswaran et al. have shown that TbRRM1 plays a complex role in mRNA regulation. In the procyclic stage, TbRRM1 is present in ribonucleoprotein complexes enriched for particular sets of mRNAs, among them, mRNAs preferentially expressed in the bloodstream stage, thus suggesting a post-transcriptional role. These authors also proposed an additional role for TbRRM1 in modulating chromatin structure, since it regulates histone occupancy in at least two regions of the genome [36].

Finally, as shown before by Gupta et al, *T*. *brucei* SR proteins seem to be implicated in both rRNA and mRNA processing, and mRNA stability [33]. The data presented in this report showing expression modulation of the nucleolar protein TbNOP86 by TbRRM1 suggests that also this SR-related protein might be somehow involved in rRNA biogenesis. In addition, our results also expand the function of this group of proteins in cell cycle control and apoptosis-like cell death, a role in which the mammalian SR proteins also participate and coordinate [16,54].

## Supporting Information

S1 Fig Cell viability assay as determined by Rho123 or Propidium Iodide (PI) incorporation. **(A)** Percentage of positive Rho123 cells, and **(B)** percentage of parasites positive for PI at different time points after *TbRRM1* silencing. Each experimental time point represents the average and respective standard deviation of at least three independent experiments. Data were analyzed by Student t test compared to control samples. *p<0.05; **p<0.01.
(TIF)

S2 Fig Micrographs of different zoid phenotypes and a lollipop parasite. **(A)** Short zoid parasite. **(B)** Long zoid cell. **(C)** Lollipop zoid parasite. **(D)** Lollipop 1N1K parasite. Scale bar = 3 μm. **(E)** Graphic showing the percentage of different zoid cell population at different time points after TET addition. Short and long zoid phenotypes were determined by measuring the distances between K-P as described. Lollipop population was determined by evaluating the parasite morphology.
(TIF)

S3 Fig Representative dot plot graphic from FACS analysis of BrdU incorporation in TbRRM1 RNAi cells from TET- or TET+ cultures. The 24 h TET- inset shows the dot plot from parasites grown in absence of BrdU.
(TIF)

S4 Fig Micrographs of BrdU incorporation assay in (A) parasites from TET- or TET+ cultures, and (B) a nozzled parasite sampled at 24 h after induction. Scale bar: 10 μm.
(TIF)

S5 Fig Detection of mitochondrial membrane potential (ΔΨm). **(A)** Flow cytometric histograms from control cells and from parasites labeled with Rho123 (FL-1) at different time points after *TbRRM1* silencing. The time-dependent reduction of Rho123 fluorescence intensity, indicates the depolarization of the mitochondrial membrane. Histograms representative of triplicate experiments are shown. **(B)** Mean relative fluorescence of Rho123 from un-induced cultures and from parasites at different time points after TET addition. **(C)** Mean relative fluorescence values ± SD of at least three independent experiments.
(TIF)

S1 Table Cell cycle distribution values. Percentage of cells in each cell cycle phase at different time points after *TbRRM1* silencing. Except for the 0 h point (unique-biological replicate), all other values correspond to the means of at least three independent experiments ± SD.
(PDF)
