# Supplementary figures and images for: Depletion of the SR-Related Protein TbRRM1 Leads to Cell Cycle Arrest and Apoptosis-Like Death in Trypanosoma brucei
Source: PLoS One. 2015 Aug 18;10(8):e0136070. doi: 10.1371/journal.pone.0136070 (PMC4540419; doi:10.1371/journal.pone.0136070)

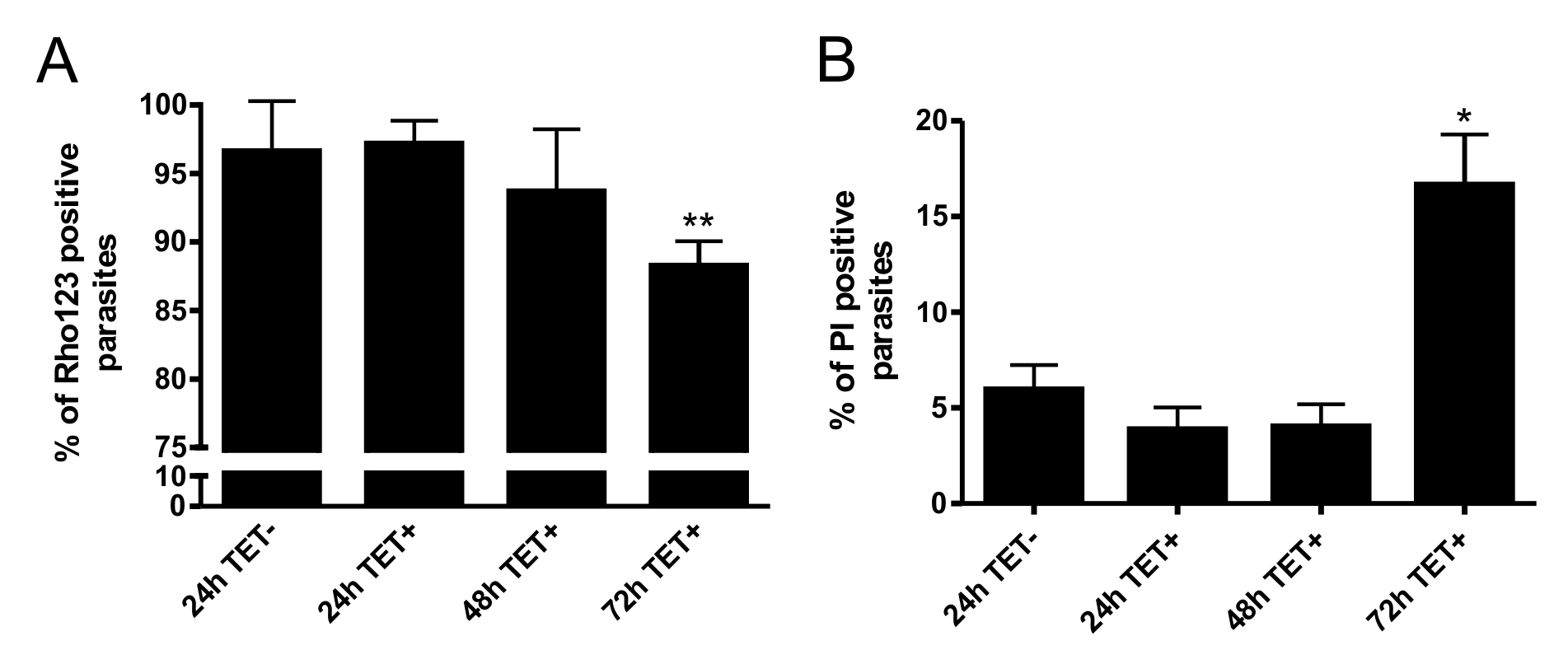

Supplement: S1 Fig — (A) Percentage of positive Rho123 cells, and (B) percentage of parasites positive for PI at different time points after TbRRM1 silencing. Each experimental time point represents the average and respective standard deviation of at least three independent experiments. Data were analyzed by Student t test compared to control samples. *p<0.05; **p<0.01. (TIF) [file pone.0136070.s001.tif]

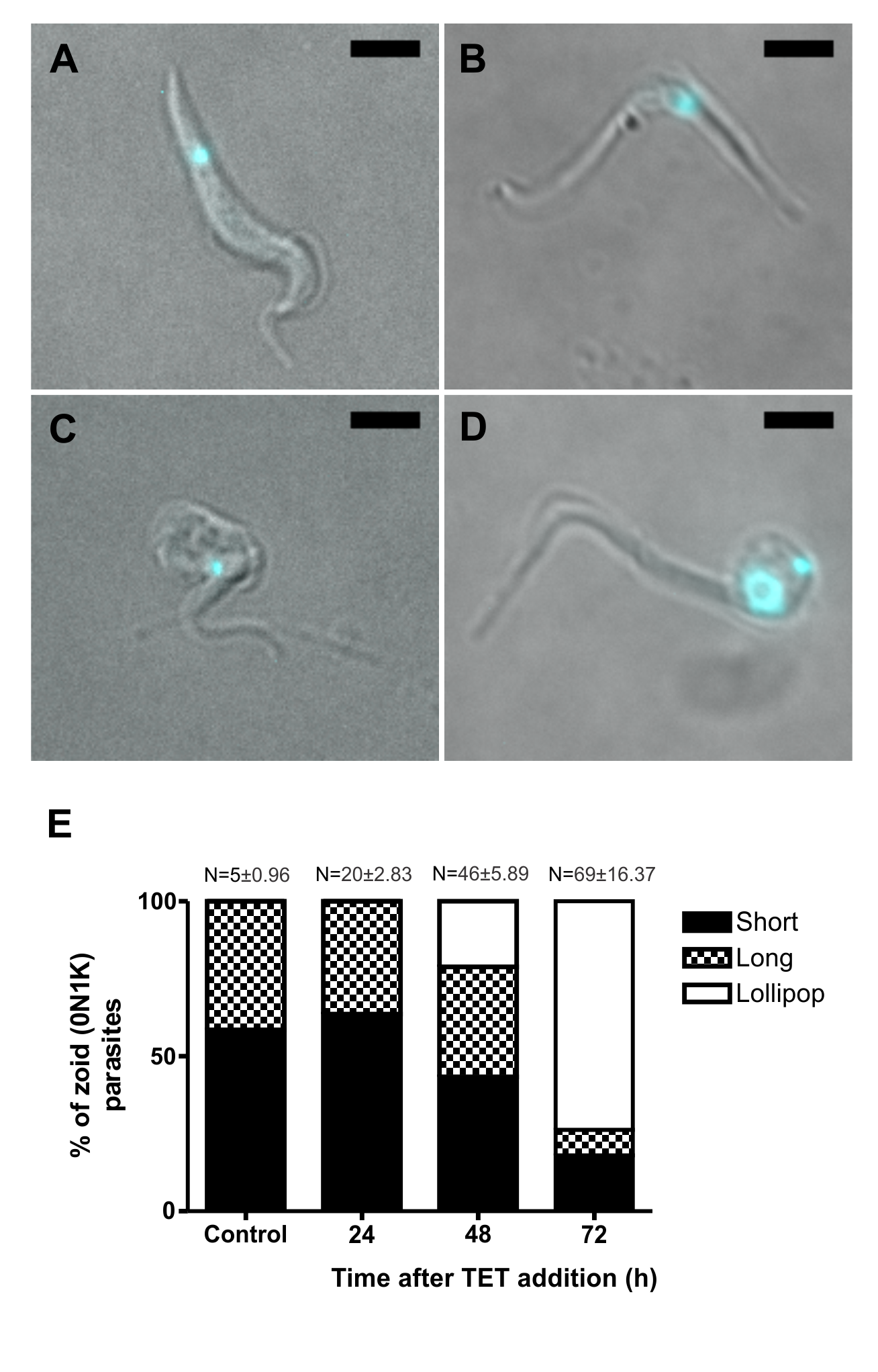

Supplement: S2 Fig — (A) Short zoid parasite. (B) Long zoid cell. (C) Lollipop zoid parasite. (D) Lollipop 1N1K parasite. Scale bar = 3 μm. (E) Graphic showing the percentage of different zoid cell population at different time points after TET addition. Short and long zoid phenotypes were determined by measuring the distances between K-P as described. Lollipop population was determined by evaluating the parasite morphology. (TIF) [file pone.0136070.s002.tif]

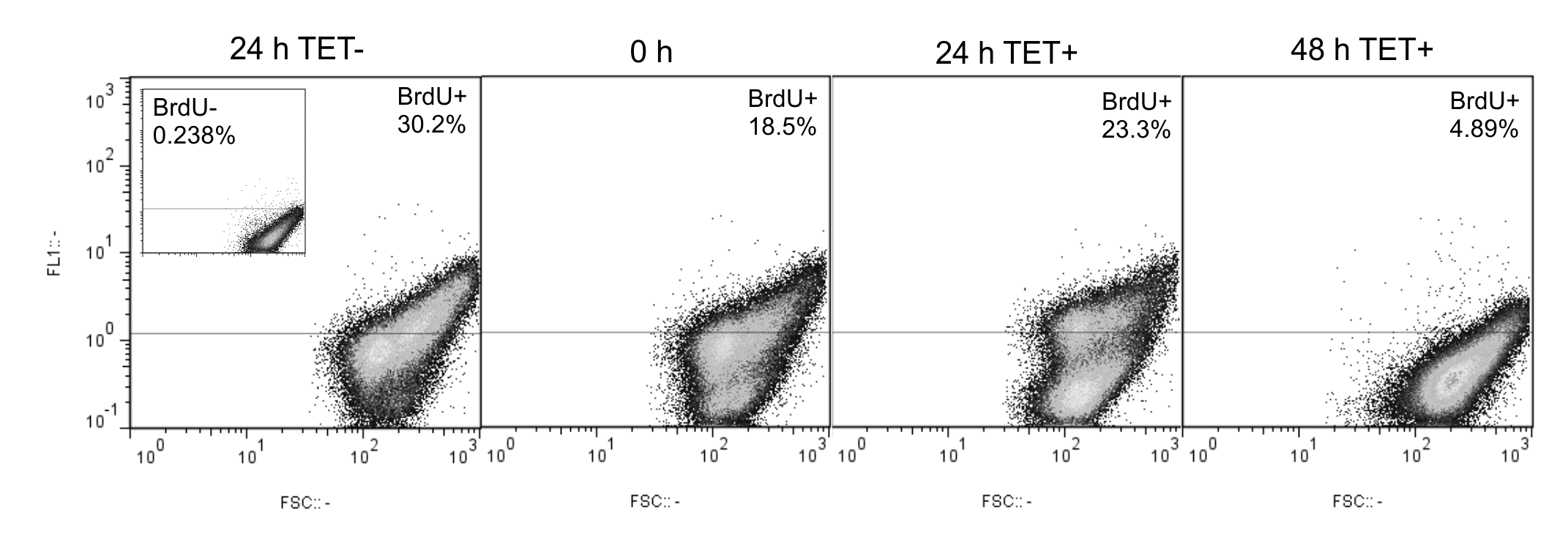

Supplement: S3 Fig — The 24 h TET- inset shows the dot plot from parasites grown in absence of BrdU. (TIF) [file pone.0136070.s003.tif]

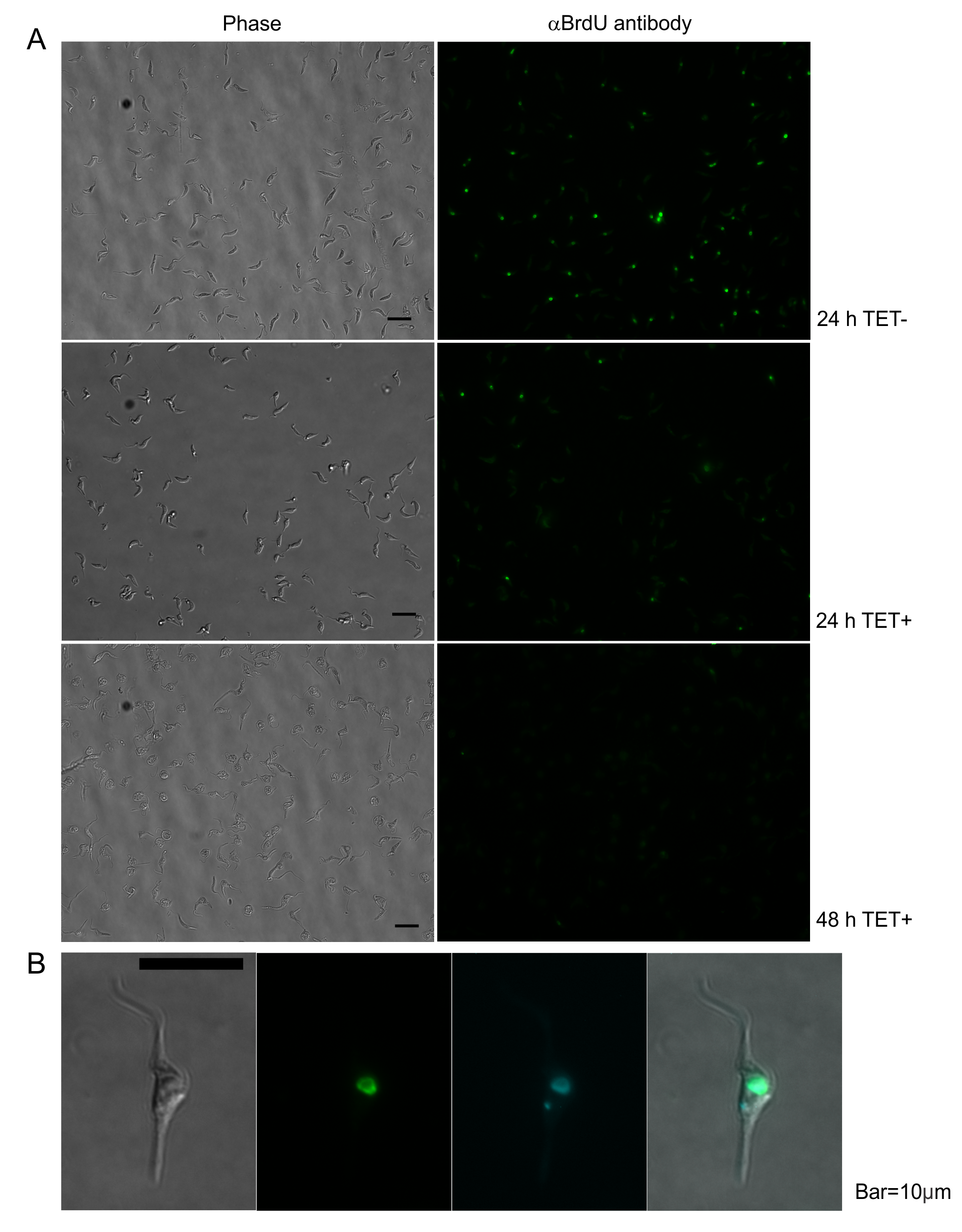

Supplement: S4 Fig — Scale bar: 10 μm. (TIF) [file pone.0136070.s004.tif]

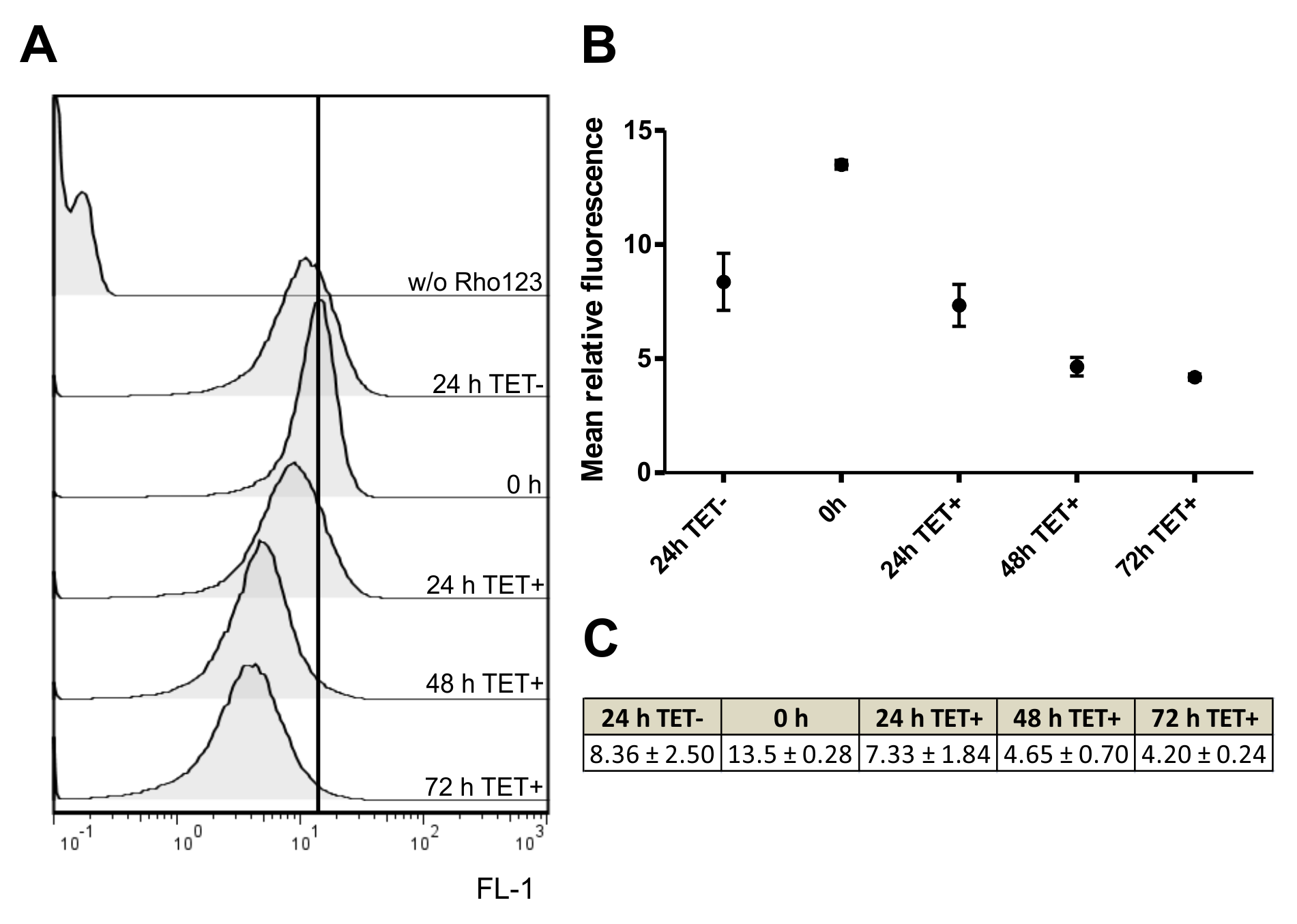

Supplement: S5 Fig — (A) Flow cytometric histograms from control cells and from parasites labeled with Rho123 (FL-1) at different time points after TbRRM1 silencing. The time-dependent reduction of Rho123 fluorescence intensity, indicates the depolarization of the mitochondrial membrane. Histograms representative of triplicate experiments are shown. (B) Mean relative fluorescence of Rho123 from un-induced cultures and from parasites at different time points after TET addition. (C) Mean relative fluorescence values ± SD of at least three independent experiments. (TIF) [file pone.0136070.s005.tif]
