# Supplementary material for: Depletion of the SR-Related Protein TbRRM1 Leads to Cell Cycle Arrest and Apoptosis-Like Death in Trypanosoma brucei
Source: PLoS One. 2015 Aug 18;10(8):e0136070. doi: 10.1371/journal.pone.0136070 (PMC4540419; doi:10.1371/journal.pone.0136070)
Supplement: S1 Table — Percentage of cells in each cell cycle phase at different time points after TbRRM1 silencing. Except for the 0 h point (unique-biological replicate), all other values correspond to the means of at least three independent experiments ± SD. (PDF) [file pone.0136070.s006.pdf]

Time	subG1	G1	S	G2/M	>G2/M
**0 h**	2.24	44.6	11.3	35.6	6.3
**24 h TET-**	1.42±0.58	46.34±1.18	11.82±0.92	32.98±4.31	7.35±3.41
**24 h TET+**	2.30±0.70	55.65±1.27	8.98±1.54	25.45±1.32	7.57±3.29
**48 h TET+**	13.5±1.06	44.83±1.19	8.66±0.24	24.77±2.42	8.23±0.15
**72 h TET+**	53.87±3.81	25.8±2.29	7.11±0.79	7.58±1.40	5.62±0.16
